# Isolation, Identification and Characterization of Growth Parameters of *Pseudomonas putida* HSM-C2 with Coumarin-Degrading Bacteria

**DOI:** 10.3390/molecules27186007

**Published:** 2022-09-15

**Authors:** Shen Huang, Menghuan Wang, Duobin Mao, Aamir Rasool, Chunxiao Jia, Pengfei Yang, Li Han, Meiling Yan

**Affiliations:** 1 College of Food and Bioengineering, Zhengzhou University of Light Industry, Zhengzhou 450001, China; 2 Institute of Biochemistry, University of Balochistan, Quetta 87300, Pakistan; 3 Anhui Tobacco Industry Co., Ltd., Hefei Cigarette Factory, Hefei 230000, China

**Keywords:** *Pseudomonas*, whole-genome sequencing, coumarin, dihydrocoumarin

## Abstract

Natural coumarins contribute to the aroma of licorice, and they are often used as a flavoring and stabilizing agents. However, coumarins usage in food has been banned by various countries due to its toxic effect. In this study, a strain of HSM-C2 that can biodegrade coumarin with high efficiency was isolated from soil and identified as *Pseudomonas putida* through performing 16S rDNA sequence analysis. The HSM-C2 catalyzed the biodegradation up to 99.83% of 1 mg/mL coumarin within 24 h under optimal culture conditions, such as 30 °C and pH 7, which highlights the strong coumarin biodegrading potential of this strain. The product, such as dihydrocoumarin, generated after the biodegradation of coumarin was identified by performing GC-MS analysis. The present study provides a theoretical basis and microbial resource for further research on coumarin biodegradation.

## 1. Introduction

Coumarin, also known as 2-H-1-chromene-2-ketone, is a flavoring agent with a peculiar fragrance that consists of an aromatic ring fused to a condensed lactone ring, and it is widely distributed in higher plants and microorganisms [1]. Due to its peculiar fragrance, coumarin has been in use in traditional spices, such as black coumarin tincture and sweet clover for centuries; therefore, now it is used as a flavoring agent and stabilizer in many industrial applications. However, the survey of pharmacovigilance in France’s results reported the hepatotoxic effect of coumarin that leads to liver failure, and it lacks the predictors of hepatotoxicity [2]. In 2017, the World Health Organization (WHO) listed coumarin as a third-grade carcinogen and severely restricted its use in food, cosmetics, and cigarettes.

The dihydrocoumarin, also known as 3,4-2H-1-chromene-2-ketone, has a coconut-like aroma, and therefore it is commonly used as a substitute of coumarin to make the flavored tobacco. The Flavor Extract Manufacturers Association (FEMA) has reported that the dihydrocoumarin is “generally considered safe” and set limits for its use in food products [3]. Only a few studies could develop physical and chemical methods suitable for removing the coumarin from extracts, but their application was limited due to their high running cost [4,5]. Microbial biodegradation is considered a green and economically-competitive alternative because microorganisms are well known for their efficient and selective catalysis of environmental hazards [6]. Therefore, many research groups are trying to develop a safe and effective method for selective degradation of coumarin into less toxic and non-carcinogenic molecules to improve the safety of food, tobacco-based products, and other products containing this type of fragrance.

The genus *Pseudomonas* was first discovered in 1894. Members of the genus *Pseudomonas* are Gram-negative bacteria, which are usually rod-shaped or slightly curved bacilli, do not form spores, have a capsule, their movement depends on one or more polar flagella, have a perfect respiratory enzyme system, can carry out aerobic respiration, and need molecular oxygen as a hydrogen acceptor to complete oxidative respiration. At present, several coumarin-degrading microbial strains such as *Arthrobacter* [7], *Pseudomonas* [8], *Saccharomyces* [9], *Aspergillus* [10], *Glomerella* [11], and *Cunninghamella* [12] have been successfully isolated. However, a very limited number of microorganisms are available for efficient degradation of coumarin and information about their genetics is limited.

In this study, a strain (*Pseudomonas putida*) with the highest efficiency degradation of coumarin into dihydrocoumarin was isolated from the soil. In addition, its growth parameters were studied and fermentation conditions were optimized, and finally annotated its functional genes.

## 2. Experimental

### 2.1. Sample Collection, Bacterial Isolation, and Selection

The HSM-C2 strain was preliminarily isolated from the soil at a depth of 5–10 cm by following the geographical coordinates: 34°81′ N, 113°50′ E. This soil was located in Zhengzhou, Henan province of China, where following cultivars such as walnuts, persimmons, and corn were grown. The collected soil samples were transferred to the laboratory for bacterial isolation and then 5 g of soil was placed in 30 mL sterile water, shaken vigorously for 1 h, and then enriched in Luria-Bertani (LB) medium at 30 °C with a 2% inoculation amount. The enriched culture was transferred to a selective medium (fermentation medium) containing coumarin as a sole carbon source at 2% inoculum for one week. The selective medium was composed of the following ingredients and taken in g/L: MgSO_4_ • 7H_2_O, 0.50; FeSO_4_ • 7H_2_O, 0.005; NaCl, 0.50; KH_2_PO_4_, 0.65; K_2_HPO_4_, 1.00; MnSO_4_, 0.001; (NH_4_)_2_ • SO_4_, 0.50; Na_2_MoO_4_ • 2H_2_O, 0.005; CaCl_2_ • 2H_2_O, 0.10; and coumarin, 1.

As compared with the control group, we observed visual and odor changes in the enrichment growth media, and the degradation of coumarin was analyzed by using gas chromatography coupled with mass spectrometry (GC–MS). Consequently, we screened a group of mixed strains containing bacteria capable of the catalysis of biodegradation of coumarin. The selected bacterial soil extract was serially diluted with sterile water from 10^−1^ to 10^−6^. The dilution was then inoculated into a selective medium supplemented with 20 mg/mL agar, at 30 °C until colonies reached a diameter of 0.5–2 mm. The isolated colony of the HSM-C2 strain was inoculated in the fermentation medium, cultured at 30 °C, and agitated at 120 rpm for 24 h. The culture supernatant was collected and degradation products of coumarin were analyzed by using GC-MS. The strain was preserved in 60% (*w*/*v*) glycerol and stored at −80 °C.

### 2.2. Evaluation of Growth Parameters and Coumarin Degradation Potential of HSM-C2 Strain

The optimal growth parameters and coumarin degradation potential of the HSM-C2 strain were evaluated. The following coumarin degradation parameters were studied: (i) initial concentration of coumarin in the fermentation medium, (ii) temperature of fermentation medium, (iii) additional carbon source and content, (iv) additional nitrogen source and content, and (v) initial pH value of the fermentation medium.

The biodegradation experiments were performed by using above mentioned fermentation medium and the HSM-C2 culture was incubated in a shaking incubator set at 120 rpm and 30 °C. The samples for construction of the growth curve and biodegradation curve were obtained by sampling out the culture at the following intervals; 0, 8, 16, 24, 32, 40, 48, 56, 64, and 72 h. Subsequently, the following single-factor experiments were performed to investigate the biodegradation potential and effect of environmental factors on biodegradation of coumarin by HSM-C2 strain and it was cultured for 20 h: (i) concentration of coumarin (2, 4, 6, 8 mg/mL); temperature (20 °C, 25 °C, 35 °C, 40 °C and 45 °C); external carbon source (fructose, glucose, lactose, maltose, β-cyclodextrin, sucrose); optimal carbon source concentration (0.5, 1, 2, 4, 6, 8 mg/mL); external nitrogen source (ammonium nitrate, yeast powder, potassium nitrate, peptone, sodium nitrate, urea); optimal nitrogen source concentration (0.5, 1, 2, 4, 6, 8 mg/mL); and initial pH (5.5, 6, 6.5, 7, 7.5, 8). The control experiment was designed and performed by following the above mentioned parameters; however, without inoculating the HSM-C2 strain.

The coumarin bioconversion by the HSM-C2 strain was studied by using the analytic instrument GC-MS. Briefly, the samples were extracted with analytical grade CH_2_Cl_2_, and then the solvent was dehydrated with anhydrous sodium sulfate and concentrated by using a rotary evaporator set at 37 °C. Subsequently, the extract was re-dissolved in 1 mL CH_2_Cl_2_ of analytical grade and filtered by using an organic filter membrane. Afterward, GC-MS analysis was performed by injecting the sample into a GC instrument equipped with column HP-5 MS 5% Phenyl Methyl Silox (size 30 m × 0.25 mm; 0.25 µm film thickness) coupled with MS having a quadrupole mass analyzer. The analytical grade helium was used as a carrier gas with a flow rate of 1 mL min^−1^. The injector and detector were set at 280 °C. The initial column temperature was maintained at 50 °C for 3 min, and then raised up to 280 °C at the rate of 4 °C min^−1^. The data were acquired in full scan modes and each mass spectrum was recorded in the *m*/*z* range 35–550 u (atomic mass units), and compared with Wiley NIST 2020 database data for compound identification. For the quantification of coumarin, an external standard method was used and the standard curve was built. The rate of coumarin degradation was found by using a formula derived from a standard curve of coumarin. The formula for calculation of the rate of coumarin degradation is as follows: rate of coumarin degradation = (reduction in coumarin/original content of coumarin) × 100%. The polar lipids were extracted and separated by two-dimensional TLC and identified by spraying with appropriate detection reagents [13,14]. The cellular menaquinones were extracted and purified as described by Collins [15], and analyzed by HPLC [16]. Non-hydroxylated fatty acids were extracted, purified, methylated, identified, and quantified by gas chromatography coupled with the standard Microbial Identification System (MIDI) [17,18].

### 2.3. Identification of Biodegradation Products of Coumarin

The inoculum of a 6 mL culture was added into a 294 mL selective medium containing 1 g/L coumarin in a 1000 mL Erlenmeyer flask, and samples were collected after following time intervals, such as 0, 8, 16, 24, 32, 40, 48, 56, 64, and 72 h, and then performed the product analysis. A control was carried out, which contained coumarin with a selective medium. The pretreatment, separation, and detection conditions of the product are same as above.

### 2.4. Identification of HSM-C2 Strain

The HSM-C2 strain was identified and characterized by performing the following techniques, such as colony morphology, cell shape, scanning electron microscope, gram staining, and physiological and biochemical characteristics determination (Table S1).

The HSM-C2 strain was identified by performing 16S rDNA sequencing. The colonies were separately grown in LB medium for 12 h, and then 1 mL bacterial culture was taken into a 1.5 mL centrifuge tube, and centrifuged at 13,000× *g* rpm for 2 min, after that the supernatant was discarded and the bacterial cell pellet was collected. The DNA was extracted by using a bacterial genomic DNA extraction kit, and universal primers 27F (5′AGA GTT TGA TCC TGG CTC AG-3′) and 1492R (5′GGT TAC CTT GTT ACG ACT T-3′) [19] were used to amplify 16S rDNA sequence and the amplification products were sent to Shanghai Parsono Biotechnology Co., Ltd. (Shanghai, China). for sequencing. The 16S rDNA sequences of *Pseudomonas* genus strains were downloaded from NCBI to perform the phylogenetic analysis of the HSM-C2 strain. The BLAST online website (https://blast.ncbi.nlm.nih.gov/Blast.cgi (accessed on 11 June 2022)) was used for sequence comparison and a maximum-likelihood method was employed to construct the phylogenetic trees [20] by MEGA software (Version 7.0, Mega Limited, Auckland, New Zealand) [21]. The 16S rDNA sequences were submitted to NCBI Sequence Read Archive (https://www.ncbi.nlm.nih.gov (accessed on 11 June 2022)) with Accession Number ON679666.

### 2.5. Whole Genome Sequencing (WGS) of Strain HSM-C2

The genomic DNA was extracted by performing the above-mentioned method. The Shanghai Parsono Biotechnology Co., Ltd. (Shanghai, China), through Illumina Hiseq 2500, sequenced the whole genome of the HSM-C2 strain. Firstly, a library of small DNA fragments (400 bp) was established, and then the quality of the library was tested. After quality testing, the library of DNA fragments was sequenced through Illumina NovaSeq. The raw data were used to control the quality of data by FastQC (Version 0.11.7, Simon Andrews, Cambridge, England) [22], and AdapterRemoval (Version 2.2.2, Mikkel Schubert, Copenhagen, Denmark) [23] was used to remove the reads with adapters and low-quality sequences. The quality of all reads is corrected based on Kmer frequency. HGAP (Version 4, Chen-Shan Chin, CA, USA) [24] and CANU (Version 1.7.1, Sergey Koren, MD, USA) [25] software were used to assemble the effective data, and Pilon software (Version 1.18, Broad institute, Cambridge, MA, USA) was used to correct the contig results of three generations. Finally, the complete sequence was obtained. GeneMarkS software (Version 4.32, Georgia Institute of Technology, Atlanta, GA, USA)was used to predict the whole genome sequence [26]. The diamond software (Version 0.8.36, Benjamin Buchfink, Tübingen, Germany) was used to annotate the genome sequences coding protein by comparing it with the corresponding database [27]. The sequence data were submitted to NCBI Sequence Read Archive with Accession Number CP100652.

## 3. Results

### 3.1. Isolation and Identification of Coumarin-Biodegrading Bacterium

**Figure 1:**
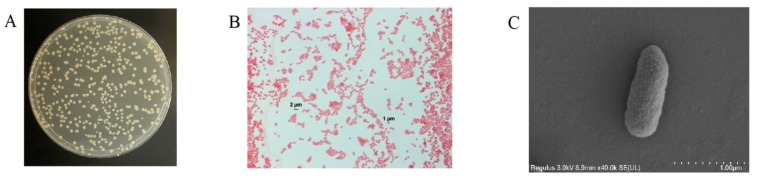
Morphological characteristics of *Pseudomonas putida* HSM-C2. (**A**) colony morphology, (**B**) gram staining result (10 × 100), (**C**) SEM result (the magnification is 40,000×).

In this study, we screened a high-efficiency coumarin-degrading bacterial strain by using coumarin as the sole carbon source. The screened strain was designated as HSM-C2 which converts the coumarin into a safe product, the dihydrocoumarin. The HSM-C2 strain possesses the following phenotypic features, such as it is Gram-negative, short, and rod-shaped (1–2 μm) and colonies on the separation medium were off-milky white, round, moist, sticky, opaque, and mycelium-free (Figure 1).

**Figure 2:**
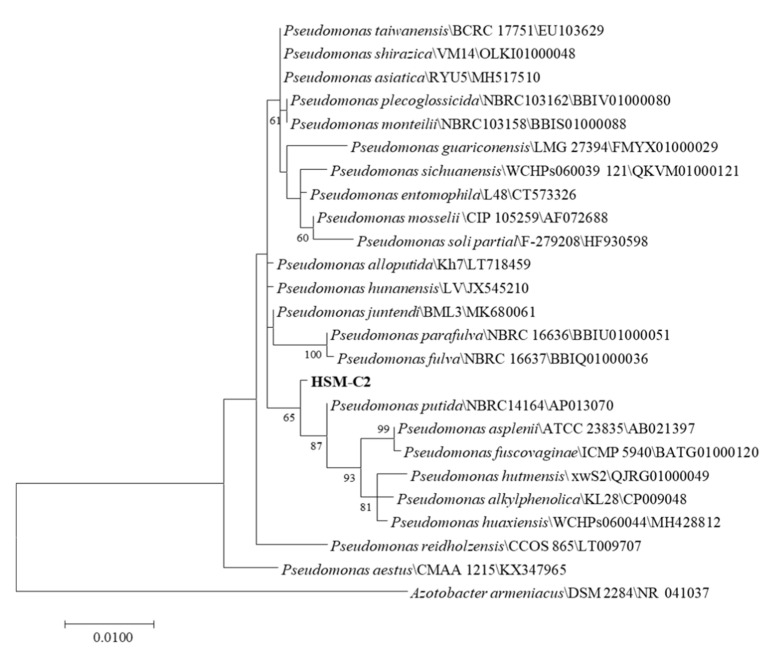
Maximum-likelihood phylogenetic tree based on the 16S rDNA gene of strain HSM-C2 and closely related strains. Bootstrap values > 50% (1000 replicates) are shown next to each branch. Filled circles indicate branches found in the phylogenetic consensus tree generated with the neighbor-joining method. Bars represent 0.01 expected changes per site.

The Blast homology comparison of sequencing results showed that the 16S rDNA length is 1456 bp. The BLAST comparison results showed that the 16S rDNA had high similarity with *Pseudomonas putida* NBRC 14164^T^ (99.8%), *Pseudomonas asiatica* RYU5^T^ (99.8%), and *Pseudomonas taiwanensis* BCRC17751^T^ (99.8%). The gene sequences with a homology greater than 98% were selected. The software MEGA (Version 7.0, Mega Limited, Auckland, New Zealand) was used for the construction and analysis of the phylogenetic trees. The phylogenetic tree was constructed by the maximum-likelihood method, and by 1000 repeated detections. The 16S phylogenetic tree (Figure 2) showed that the HSM-C2 strain formed a distinct phyletic line with *P. putida* [28,29], and therefore it belongs to *P. putida* of *Pseudomonas*, but it is located on a separate branch of phylogenetic trees. We have also performed the preliminary identification of *Pseudomonas putida* by employing molecular, physiological, and biochemical methods.

**Figure 3:**
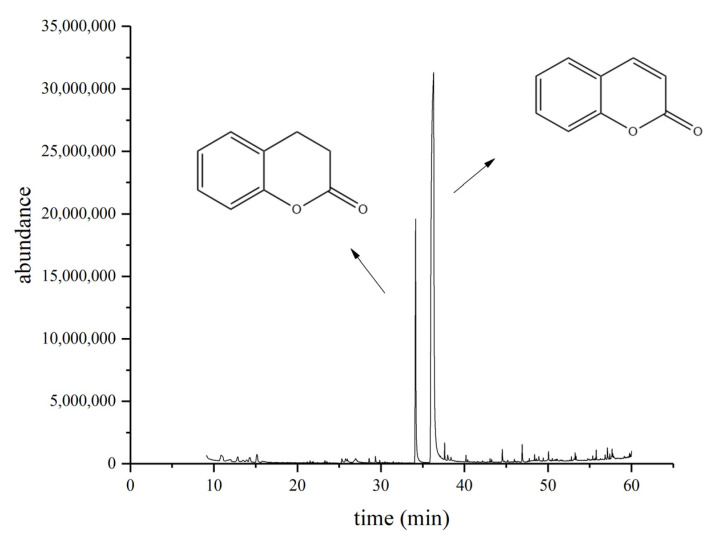
GC-MS analysis of supernatant from fermentation media of HSM-C2 strain showed that the coumarin content decreased with concomitant production of dihydrocoumarin.

The coumarin biodegradation capability of the HSM-C2 strain was confirmed by performing the GC-MS analysis, which displayed that the concentration of coumarin was significantly reduced with the concomitant production of dihydrocoumarin in the growth medium containing the HSM-C2 strain compared with the growth medium without the HSM-C2 strain (Figure 3). Therefore, the ability of the HSM-C2 strain to biodegrade the coumarin may be used as a typical feature to differentiate it from closely related strains such as *Pseudomonas* strains.

### 3.2. Determination of Growth Curve and Degradation Curve of HSM-C2 Strain

The coumarin was quantified by the external standard method and the standard curve was built by following a six-point calibration curve, and the R^2^ was above 0.99 (Figure S1), which indicates that the standard curve is accurate, and it can be used for the calculation of coumarin.

**Figure 4:**
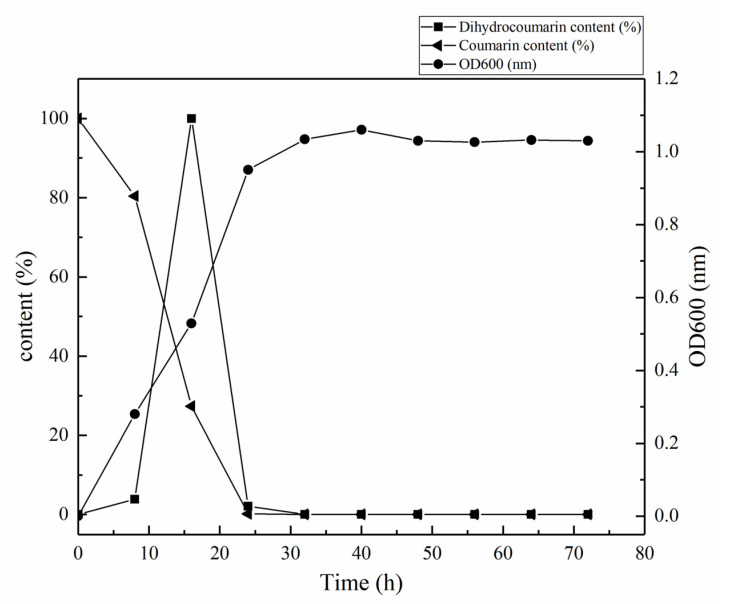
Growth curves of strain HSM-C2 and the content curves of coumarin and dihydrocoumarin.

The growth curve of the HSM-C2 strain was constructed via recording optical density (OD_600_). It was observed that the HSM-C2 strain grew slowly during 1–12 h compared with control due to its adaptation to the coumarin-containing growth media. After adaption, the HSM-C2 strain grew exponentially fast and produced dihydrocoumarin from coumarin; therefore, the content of coumarin exponentially decreased during later hours of the growth period (Table S2, Figure 4). Specifically, the HSM-C2 strain quickly degraded the coumarin during the logarithmic growth phase (8–24 h), and the degradation rate reached up to 99.83% at 24 h, which can be confirmed by the decreased concentration of coumarin and reciprocal accumulation of dihydrocoumarin in the culture media (Table S2, Figure 4). Moreover, the coumarin content of the fermentation media and optical density of the HSM-C2 strain reached an equilibrium state at the 24th hour of the fermentation period (Table S2, Figure 4). The conversion of coumarin into dihydrocoumarin reached the maximum level at the 16th hour of the fermentation period.

### 3.3. Determination of Effect of Environment Factors Biodegradation Process

**Figure 5:**
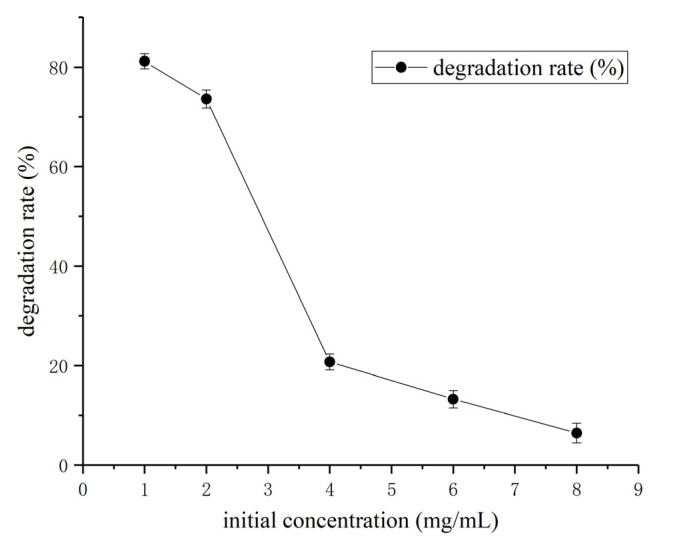
Effect of initial coumarin concentration on coumarin degradation rate. The results are expressed as the mean ± SD from three independent experiments.

The HSM-C2 strain displayed the highest coumarin degradation rate at 1 mg/mL concentration of coumarin, whereas increments in the initial concentration of coumarin resulted in a reciprocal decrease in the degradation ability of HSM-C2 strain (Figure 5). The results demonstrate that the ideal concentration for the growth of the HSM-C2 strain on coumarin is 1 mg/mL and an increase in its concentration has an inhibitory effect.

**Figure 6:**
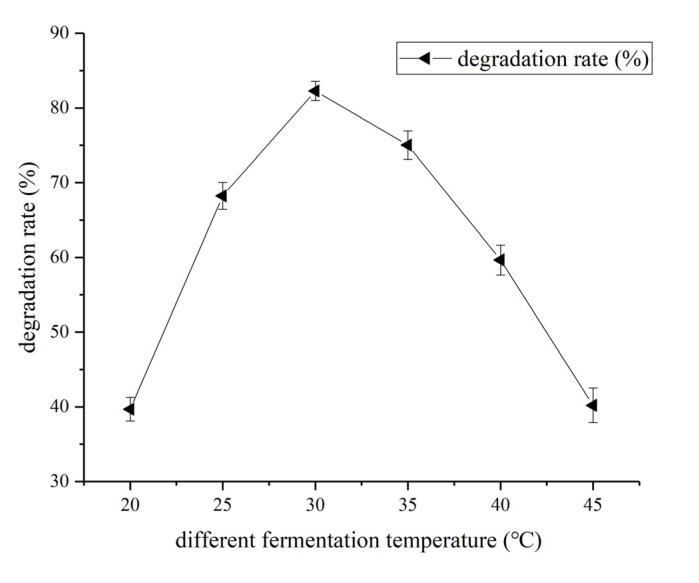
Effect of fermentation temperature on coumarin degradation rate. Data are expressed as mean ± SD of three independent experiments.

The optimal coumarin degradation temperature was screened by growing the HSM-C2 strain in the fermentation media containing 1 mg/mL coumarin and at temperatures ranging from 25 °C–45 °C (Figure 6). The highest coumarin degradation rate was recorded at 30 °C and subsequent fermentation experiments were performed at the same temperature.

**Figure 7:**
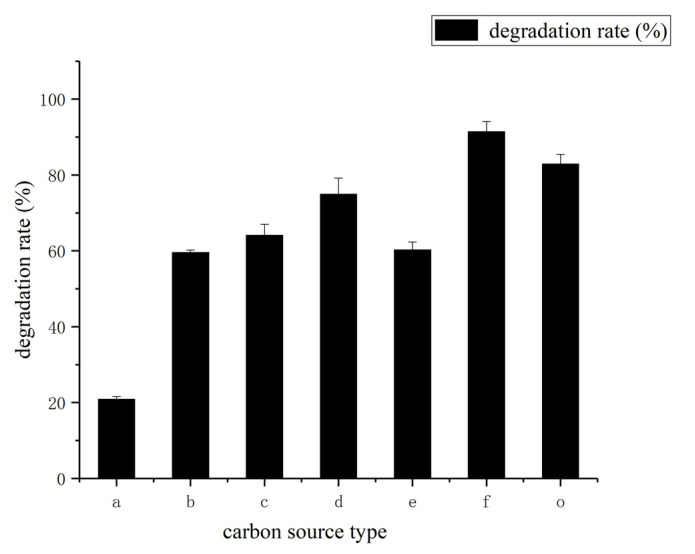
Effects of type of carbon source on coumarin degradation rate (a-glucose, b-fructose, c-malt dust, d-sucrose, e-lactose, f-β- cyclodextrin, o-contrast). The results are expressed as mean ± SD from three independent experiments.

The effect of different carbon sources such as glucose, fructose, malt dust, sucrose, lactose, and β-cyclodextrin was determined by growing the HSM-C2 strain in media containing 1 mg/mL coumarin and at a temperature of 30 °C. The *Pseudomonas* *putida* was used as a control strain to study the effect of different carbon sources (Figure 7). The results indicated that the use of β-cyclodextrin as a carbon source along with coumarin strongly improved the degradation rate of coumarin (Figure 7).

**Figure 8:**
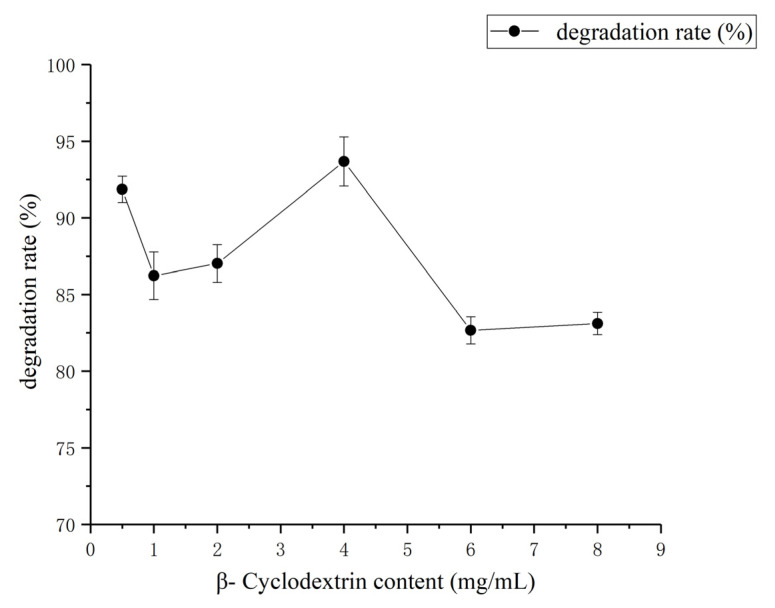
Effect of β-cyclodextrin content on coumarin degradation rate. The results are expressed as the mean ± SD from three independent experiments.

However, we could not observe any significant correlation between the effects of different doses, such as 0.5 to 4 mg/mL β-cyclodextrin, on the degradation rate of coumarin (Figure 8). Instead, the dose increment of β-cyclodextrin inflicted a negative impact on the degradation rate of coumarin and the optimal concentration of β-cyclodextrin was recorded as 0.5 mg/mL.

**Figure 9:**
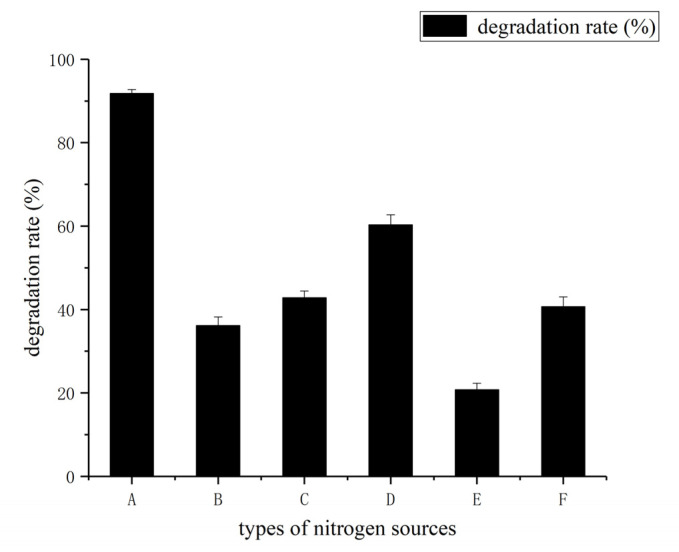
Effect of different nitrogen sources on coumarin degradation rate (A-ammonium nitrate, B-sodium nitrate, C-potassium nitrate, D-yeast powder, E-peptone, F-urea). The results are expressed as the mean ± SD from three independent experiments.

The addition of organic and inorganic sources, such as ammonium nitrate, sodium nitrate, potassium nitrate, yeast powder, peptone, and urea affected the degradation rate of coumarin by the HSM-C2 strain (Figure 9). However, the addition of ammonium nitrate in the fermentation medium of the HSM-C2 strain strongly enhanced the degradation rate of coumarin compared with others and without nitrogen sources. The result indicates that the addition of ammonium nitrate promoted the uptake of nutrients from fermentation media by *Pseudomonas putida* (HSM-C2).

**Figure 10:**
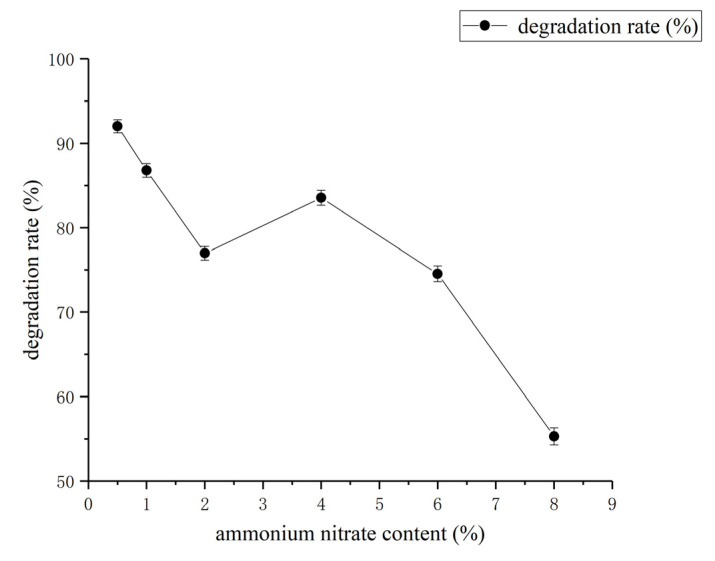
Effect of ammonium nitrate content on coumarin degradation rate. The data are presented as the mean ± SD of three independent experiments.

From Figure 10, it can be seen that the degradation rate of coumarin was highest when a 0.5 mg/mL concentration of ammonium nitrate was used in the fermentation medium and it was taken as an optimal concentration of ammonium nitrate for subsequent experiments.

**Figure 11:**
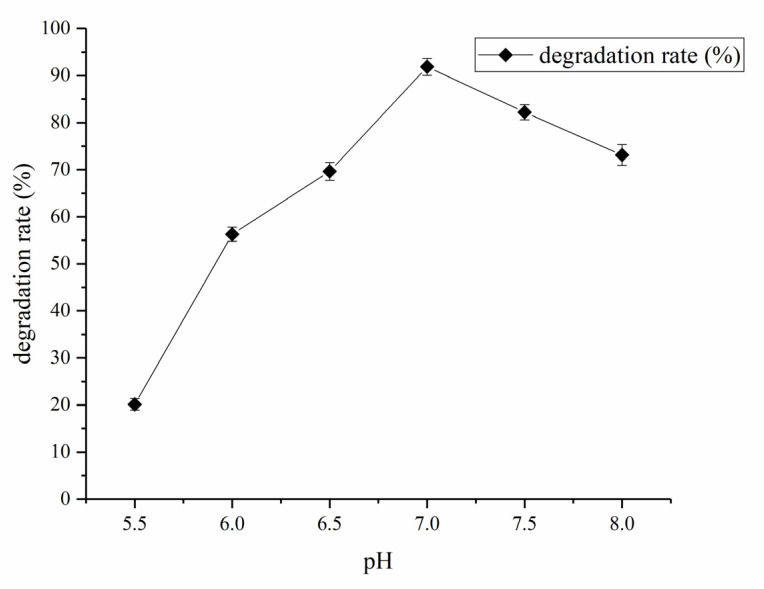
Effect of different pH values on the degradation rate of coumarin. The results are expressed as the mean ± SD from three independent experiments.

The effect of pH from pH 5.5 to 8 on the degradation rate of coumarin was also observed, and it was found that the coumarin degradation rate was increased until the pH value reached up to 7, and then it was decreased with a further increase in pH of the fermentation medium (Figure 11). As a result, pH 7 was selected as an optimal concentration for the preparation of the fermentation medium and growth of the HSM-C2 strain.

### 3.4. Sequencing and Analysis of the Whole Genome of Pseudomonas putida HSM-C2

The genome sequencing of the HSM-C2 strain was performed by using Illumina NovaSeq and Oxford Nanopore ONT (Shanghai, China). The characteristics of the genome of the HSM-C2 strain are shown in Table S3. The genome sequencing data were assembled into a complete, circular chromosome (Figure S2), which is 5,568,427 bp in size and comprised of 61.58% GC content. The genome of the HSM-C2 strain encodes 5025 proteins, 75 tRNAs, 23 rRNAs, and 81 ncRNAs. The total length of protein-coding genes is 4,920,855 bp, accounting for 88.37% of the genome length. Furthermore, the protein-coding sequences were further annotated by using NR, COGs, KEGGs, GO, and Swiss-Prot and their accession numbers are 4916, 4480, 2787, 3799, and 3755, respectively.

In total, 5025 genes were classified into 4480 COGs, of which amino acid transport and metabolism, energy production and conversion, and transcription were the most enriched COGs (Figure S3). According to the genome sequence analysis and annotation (Supplementary S2), there were 22, 13, 2, and 2 coding sequences (CDSs) encoding the NAD (P)-dependent oxidoreductase, dioxygenase, fumarylacetoacetate (FAA) hydrolase family and 3-(3-hydroxy-phenyl)propionate hydroxylase, respectively.

Importantly, based on a BLAST search, it was found that there were three continual genes, which were annotated as 3-(3-hydroxy-phenyl)propionate hydroxylase (gene_3127, 527 bp genes encoded 519 proteins) and glyoxalase bleomycin resistance protein (Dioxygenase (gene_3128, 561 bp genes encoded 186 amino acid proteins). The fumarylacetoacetate hydrolase family protein (gene_3129, 852 bp genes encoded 283 amino acid proteins) had the function of biodegradation aromatic compounds. Protein sequence alignment revealed that the products of three genes exhibited relatively high identity with the induced proteins encoded by a gene cluster hcdABC from 7-hydroxycoumarin-degrading *Pseudomonas mandelii* 7HK4 (DSM 107615) [30], and the identity percentage was 80.41% sequence identity with flavin-binding hydroxylase (HcdA), 87.63% with extradiol dioxygenase (HcdB), and 84.81% with putative hydroxymuconic semialdehyde hydrolase (HcdC).

## 4. Discussion

In the present study, a promising bacterial strain of HSM-C2, capable of degrading the coumarin, was successfully isolated and identified as *Pseudomonas putida* by cell morphology, physiological, and biochemical analysis, and 16S rDNA sequencing. *P. putida* has been reported to perform many ecological functions, including the biodegradation of environmental pollutants [31,32,33,34]. The *Pseudomonas putida* displayed an excellent coumarin biodegradation potential, which is evident from its ability to degrade nearly 100% coumarin (1 mg/mL) within 24 h higher than *Saccharomyces cerevisiae* DSMZ 2155, *Bacillus cereus*, and *Pseudomonas orientalis* (when the initial concentration of coumarin was 400 mg/L in the fermentation medium, then the coumarin degradation was 100% within 120 h, 50% within 150 h, and 40% within 150 h, respectively) [9]. The *Pseudomonas* USTB-Z has been previously reported to degrade 100% coumarin within 48 h, when the initial concentration of coumarin was 800 mg/L [6], this indicates that *Pseudomonas putida* is an efficient coumarin-degrading bacterial strain.

Subsequently, the growth rate and coumarin-degradation rate of *Pseudomonas putida* were studied. The results showed that the degradation rate of coumarin was up to 99.83% after 24 h of the fermentation period, and coumarin concentration in the fermentation medium reached an equilibrium with a number of cells of *Pseudomonas putida*. The effect of the different growth conditions on the degradation rate of coumarin by *Pseudomonas putida* was also studied and optimal growth conditions were discovered for optimal coumarin degradation by *Pseudomonas putida*. The results showed the following optimal growth conditions for optimal degradation of coumarin by *Pseudomonas putida:* the initial concentration of coumarin was 1 mg /mL, the initial pH was 7, carbon source was 0.5 mg/mL β-cyclodextrin, nitrogen source was 0.5 mg /mL ammonium nitrate, and the strain was grown at 30 °C for 24 h.

The genome-wide sequencing of *Pseudomonas putida* revealed that 39 key enzymes are involved in the degradation of coumarin (Supplementary S2). The degradation products of coumarin by other *Pseudomonas* sp. are oxalic acid and 2, 3- dihydroxyphenylpropionic acid [35]. The double bond of a pyrogen ring of coumarin is reduced by coumarin reductase, which uses NADH as a coenzyme and produces dihydrocoumarin, which is then hydrolyzed into melilotic acid [8]. Similarly, Kosuge and Con postulated that in higher plants, such as Melilotus alba, coumarin is reduced to dihydrocoumarin and then a pyrone ring is opened to form melilotic acid [36].

The possible coumarin degradation pathway in *Pseudomonas putida* is the reduction in coumarin into dihydrocoumarin by coumarin reductase, and we found no other product during 0–72 h of fermentation timeline. However, three enzymes in the whole genome of *Pseudomonas putida* strain were found highly similar to enzymes of a pathway involved in the metabolism of 3-(2, 4-dihydroxyphenyl)-propionic acid [30].

This study provides a theoretical basis and microbial resource for further exploring the degradation mechanism of coumarin.

## 5. Conclusions

*Pseudomonas putida* HSM-C2 is a bacterial strain that efficiently degrades coumarin into dihydrocoumarin. This strain biodegraded nearly 100% of 1 g/L coumarin within 24 h of the fermentation period. The optimal growth conditions for the *Pseudomonas putida* HSM-C2 strain were 30 °C, pH 7, 0.5 g/L β-cyclodextrin, and 1 g/L coumarin. The biodegradation products of coumarin were screened using GC-MS, and dihydrocoumarin was identified as the sole product. Importantly, we have initially explored many genes encoding enzymes involved in the coumarin biodegradation through whole genome sequencing.

## Data Availability

The data presented in this study are available on request from the corresponding author.

## Supplementary Materials

The following supporting information can be downloaded at: https://www.mdpi.com/article/10.3390/molecules27186007/s1, Figure S1: Calibration curve of standard of coumarin; Figure S2: The circular diagram of genome of HSM-C2 strain; Figure S3: COG function classification diagram; Table S1: Physiological and biochemical characteristics of HSM-C2 strain; Table S2: OD600 nm and coumarin content during fermentation of HSM-C2 strain; Table S3: Characteristics Genome of HSM-C2 (Supplementary S1); Supplementary S2.

## Sample Availability

Samples are available from the authors.
