# Supplementary material for: Isolation, Identification and Characterization of Growth Parameters of Pseudomonas putida HSM-C2 with Coumarin-Degrading Bacteria
Source: Molecules. 2022 Sep 15;27(18):6007. doi: 10.3390/molecules27186007 (PMC9506499; doi:10.3390/molecules27186007)
Supplement: Supplementary file 1 [file molecules-27-06007-s001.zip › Supplementary S1.pdf]

Supplementary material

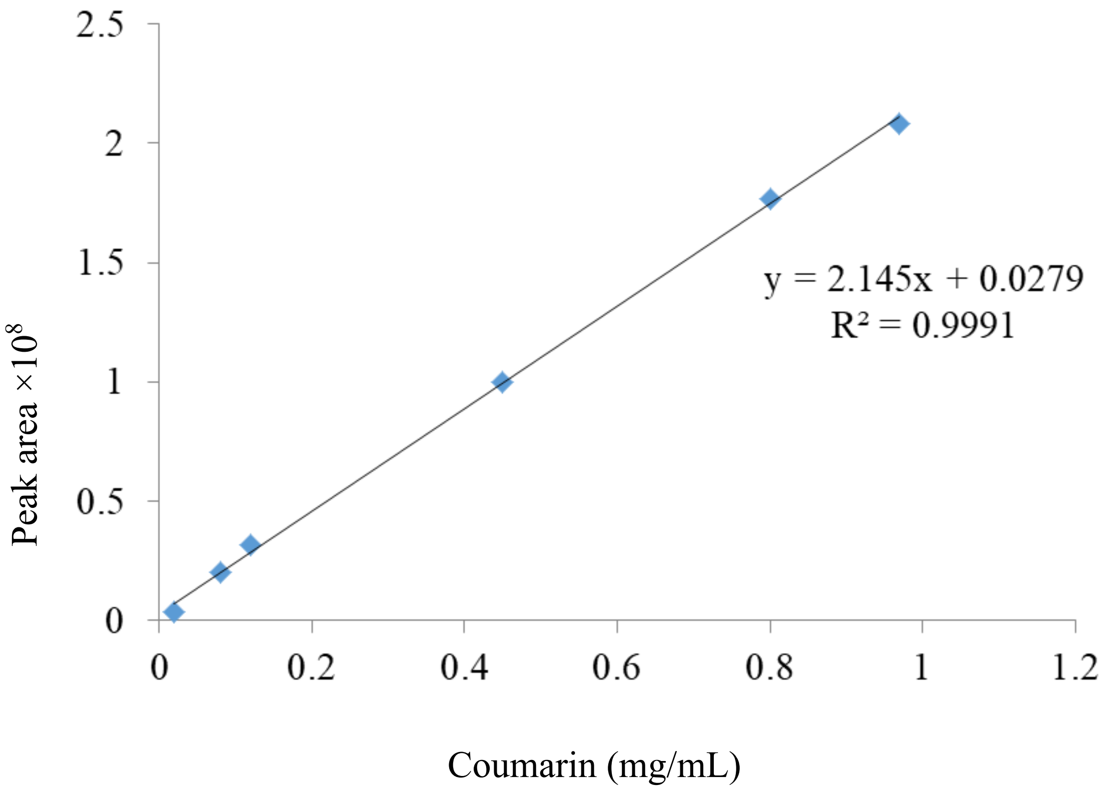

Figure S1. Calibration curve of standard of coumarin

Table S1. Physiological and biochemical characteristics of HSM-C2 strain

Features	1
pH 6.0	+
pH 5.0	-
1% NaCl	+
4% NaCl	w
8% NaCl	-
glycine-L-proline	-
L-arginine	+
L-aspartic acid	+
L-glutamic acid	w
L-histidine	w
L-serine	+
L-pyroglutamic acid	-
D-galacturonic acid	+
adipic acid	-
D-gluconic acid	+

glucuronamide	+
N-acetyl glucosamine	-
mucic acid	+
Twain 40	+
glycerin	+
L- arabinose	+
D- ribose	+
D- sucrose	-
D- xylose	+
D- side marigold	+
D- galactose	+
D- glucose	+
D- fructose	+
D- mannose	+
D- mannitol	+
honey disaccharide	+
D- xylose	+
D- fucose	+
potassium gluconate	+
2-ketogluate potassium	w
alkaline phosphatase	+
esterase (C4)	+
lipoesterase (C8)	+
arylamine leucine	+
arylamine valine	+
acid phosphatase	+
naphthol- AS-BI- phosphate hydrolase	+
cystamine arylaminase	+
N-Acetyl-β Glucosaminidase	-

-, negative; +, positive; w, weakly positive.

Table S2. OD600 nm and coumarin content during fermentation of HSM-C2 strain

Fermentation time	OD600nm	Coumarin content after fermentation/100%
0	0.000	100
8	0.280	80.3989
16	0.529	27.3587
24	0.950	0.1680
32	1.034	0.0360

40	1.060	0.033
48	1.030	0.029
56	1.026	0.028
64	1.032	0.028
72	1.030	0.028

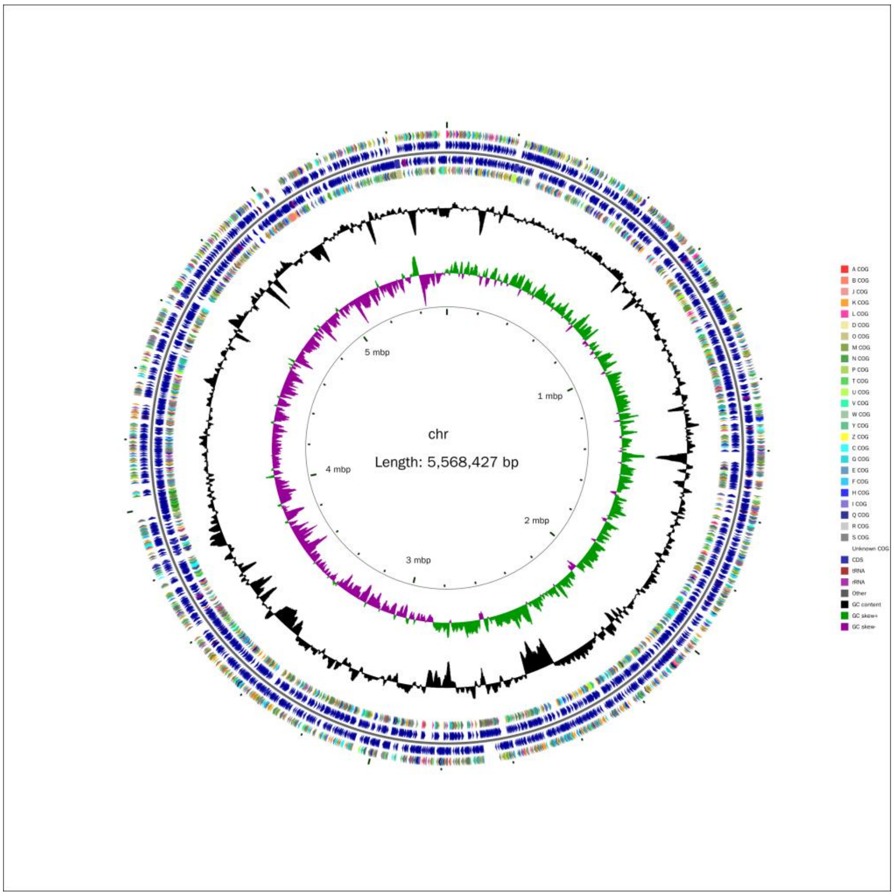

Figure S2. The circular diagram of genome of HSM-C2 strain

Table S3. Characteristics Genome of HSM-C2

**Genome Characteristics**	
Total length (bp)	5,568,427
GC content (%)	61.58

Number of protein-coding genes (bp)	5025
Average length of protein-coding genes (bp)	979.27
% of Genome (protein-coding genes)	88.37
tRNA genes	75
rRNA genes	22
ncRNA genes	81
NR	4916
COG	4480
KEGG	2787
GO	3799
Swiss-Prot	3755

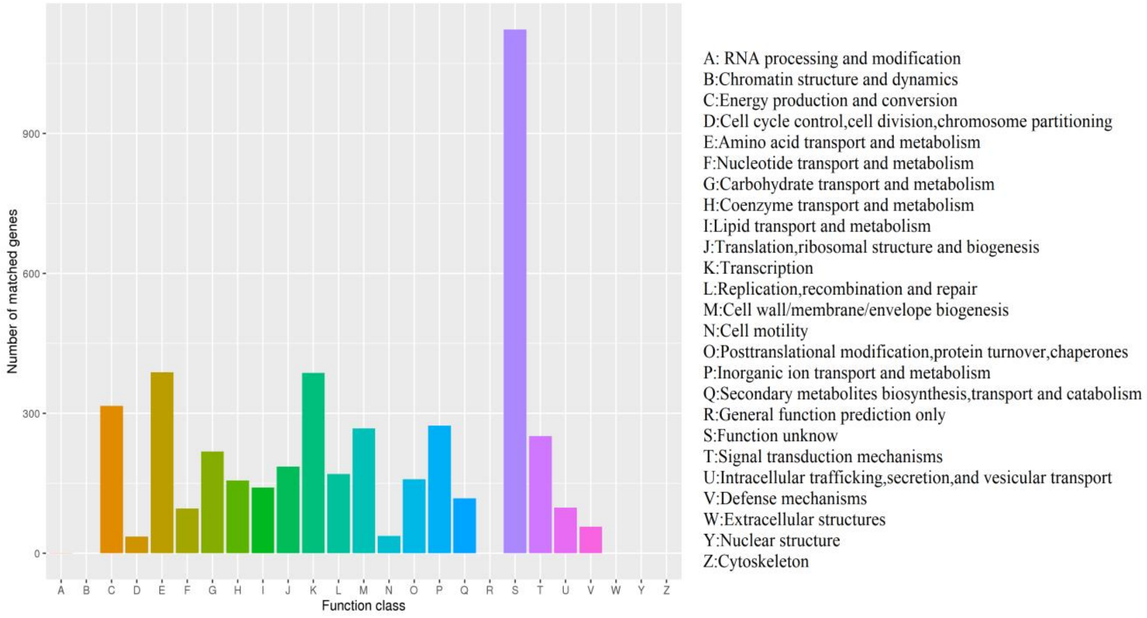

Figure S3. COG function classification diagram
